# *Plasmodium* parasites of birds have the most AT-rich genes of eukaryotes

**DOI:** 10.1099/mgen.0.000150

**Published:** 2018-01-23

**Authors:** Elin Videvall

**Affiliations:** Department of Biology, Lund University, Sweden

**Keywords:** GC-content, AT-bias, *Plasmodium*, genome evolution

## Abstract

The genomic architecture of organisms, including nucleotide composition, can be highly variable, even among closely-related species. To better understand the causes leading to structural variation in genomes, information on distinct and diverse genomic features is needed. Malaria parasites are known for encompassing a wide range of genomic GC-content and it has long been thought that *Plasmodium falciparum*, the virulent malaria parasite of humans, has the most AT-biased eukaryotic genome. Here, I perform comparative genomic analyses of the most AT-rich eukaryotes sequenced to date, and show that the avian malaria parasites *Plasmodium gallinaceum*, *P. ashfordi*, and *P. relictum* have the most extreme coding sequences in terms of AT-bias. Their mean GC-content is 21.21, 21.22 and 21.60 %, respectively, which is considerably lower than the transcriptome of *P. falciparum* (23.79 %) and other eukaryotes. This information enables a better understanding of genome evolution and raises the question of how certain organisms are able to prosper despite severe compositional constraints.

## Data Summary

Supplementary data is available with the online version of this article.

**Impact Statement**

With the advent of high-throughput sequencing, we have gained novel insights into how highly variable the genome architecture of organisms can be. Some species have small, gene-dense genomes with high AT-content, while others have repeat-ridden, GC-rich genome sequences. Describing this variation and finding organisms exhibiting extreme patterns are crucial in order to understand the processes driving genome evolution. In this study, I perform comparative analyses of the most AT-biased eukaryotes sequenced to date, and show that three recently sequenced malaria parasites of birds have the most AT-rich coding sequences by a wide margin. The remarkable compositional and functional constraints these avian parasites are subjected to, engender further questions and make these lineages particularly useful for the study of genome architecture.

## Introduction

Genomes constitute highly-dynamic landscapes which can exhibit both structural and physical variation, and their compositional architecture can have major impacts on evolutionary processes. A major challenge in evolutionary genomics has been to explain the substantial variation in the genomic guanine+cytosine (GC) content observed across species. The mean GC-content of eukaryotic micro-organisms varies drastically, with parasites commonly displaying a tendency towards evolving GC-poor genomes. GC-content is highly associated with several genomic features, such as gene density, proteome size, chromosomal region, distribution of repeat elements, and methylation patterns [1–3]. It has also been significantly correlated with recombination rate and gene expression levels [4–6]. The biological relevance of genomic GC-content therefore raises important questions regarding its evolutionary causes and consequences.

DNA sequences with higher GC-content are thermally more stable than sequences with higher adenine+thymine (AT) content because GC-pairs have an additional hydrogen bond relative to AT-pairs. As a result, theory has predicted that high genomic GC-content might be selected for in environments with high temperature. In light of this, GC-rich isochores interspersed in the genome sequences of warm-blooded vertebrates were proposed to have evolved as an adaptation to body temperature [7]. The thermal adaption hypothesis has, however, been rejected by several studies [8, 9], and analyses of whole-genome sequence data found little evidence for the isochore proposition [10]. Nonetheless, some evidence seems to suggest that the GC-content of structural RNA positively correlates with optimal temperature of prokaryotes [8, 9], lending some credibility to the theory.

Interspecific differences in codon usage depend mostly upon the compositional constraints of the genomes, as organisms with extreme nucleotide biases are heavily constrained with regards to their amino acid usage. The reasons why certain organisms evolve extreme nucleotide biases have been debated for decades [5, 11–14]. One of the main underlying mechanisms believed to be driving increased genomic GC-content is GC-biased gene conversion during recombination [5, 15]. This conversion results from mismatch repair mechanisms that are GC-biased [16–18] and leads to higher GC-content in genomic regions subjected to high recombination rates. A process proposed to be a main driver for increased AT-content is AT-biased mutations [19], such as the spontaneous deamination of 5-methylcytosine to thymine. It has been demonstrated in bacteria that mutations are universally biased towards AT, and that selection is therefore acting upon genome sequences to increase GC-content [20, 21]. Intracellular eukaryotic microbes could, in theory, evolve towards AT-richness as a result of reduced recombination rates, loss of DNA repair mechanisms, higher nucleotide substitution rates, relaxed selection pressures, increased selection for AT mutations, or a combination thereof.

An organism that repeatedly has been highlighted as having the most extreme eukaryotic genome sequence due to its low genomic GC-content (19.34 %) is the human malaria parasite *Plasmodium falciparum* [4, 22, 23]. In contrast, the related human malaria parasite *Plasmodium vivax*, exhibits a much higher genomic GC-content of 42.28 % [24]. This enormous variation in GC-content in the genus *Plasmodium* has significantly complicated comparative and phylogenetic analyses [25] and has sparked questions regarding its origin [19, 26]. Recent evidence seems to suggest that the ancestral *Plasmodium* genome was indeed AT-rich, and that *P. vivax* has managed to restore its GC-content to more normal levels [27, 28]. The underlying causes why *Plasmodium* parasites, in particular, exhibit such dynamic nucleotide compositions remain to be elucidated [29].

In this study, I perform comparative analyses of the nucleotide composition of all sequenced eukaryotes with extreme AT-bias in their transcript and coding sequences (CDS) and compile a list of the most AT-rich eukaryotes sequenced to date. I further investigate patterns of GC-content in different gene categories in the seven eukaryotes with the most AT-rich genes (*Plasmodium* spp.), and finally, I evaluate the amino acid composition and codon usage of the most extreme organisms in terms of CDS AT-bias, the avian malaria parasites.

## Methods

### Organisms

Genome and transcriptome sequences of a wide range of eukaryotic species containing the lowest GC-content sequenced to date were identified via extensive literature and database searches. The sequences were downloaded, analysed, and the most AT-rich eukaryotic organisms are described in Table 1. The introns and intergenic regions of AT-rich genomes always display lower GC-content relative to the coding sequences, mainly due to stronger negative selection pressure on coding sequences to remain functional [11]. The difficulties in comparing intergenic repetitive DNA across species, and the direct functional relevance of coding regions, are why GC comparative analyses normally focus solely on CDS [27], as I do in this study. The transcriptome of *P. ashfordi* was derived from the annotated published transcriptome assembly [30] as no genome sequence was available for this organism. Genome, transcriptome, and coding sequences for all other species of the genus *Plasmodium* were downloaded from PlasmoDB release 32 [31]. The following *Plasmodium* lineages were used: *P. falciparum* 3D7, *P. berghei* ANKA, *P. chabaudi chabaudi*, *P. gaboni* SY75, *P. gallinaceum* 8A, *P. reichenowi* CDC, *P. relictum* SGS1-like (DONANA05), *P. vivax* Sal1, *P. vinckei vinckei*, *P. yoelii yoelii* 17X and *P. ashfordi* GRW2 [22, 24, 30, 32–34]. The organism *P. gaboni* was included in the analyses due to its extraordinarily low GC-content, but note that this *Plasmodium* lineage has yet to achieve taxonomical species status [35]. Genomic datasets of the following non-*Plasmodium* lineages were downloaded from AmoebaDB release 32 [36]: *Entamoeba nuttalli* P19 and *Entamoeba dispar* SAW760, from MicrosporidiaDB release 32 [36]: *Anncaliia algerae* PRA109 [37] and *Nosema ceranae* BRL01 [38], from Ciliate.org [39]: *Tetrahymena thermophila* (v. June2014) and *Tetrahymena elliotti* (v. Oct2012) [40]. All other sequences have been obtained from their public repositories, including *Hepatospora eriocheir* and *Enterocytozoon hepatopenaei* [41], *Dictyostelium discoideum* (v. 2.7) [42], *Pecoramyces ruminantium* (*Orpinomyces* sp. C1A) [43], *Nosema apis* BRL01 [44], and *Strongyloides ratti* [45]. According to the list of sequenced genomes at The National Center for Biotechnology Information (NCBI), the eukaryote with the most AT-rich genome as of May 2017 is the protist *Ichthyophthirius multifiliis* (a ciliate parasite of fish, GC Transcripts: 24.18 %, CDS: 24.41 %, Non-CDS: 13.70 %, Genome: 15.96 %) [46], followed by *Pseudocohnilembus persalinus* (another ciliate parasite of fish, CDS: 25.19 %, Non-CDS: 14.67 %, Genome: 18.81 %) [47]. Both genome sequences of *I. multifiliis* and *Ps. persalinus* have, however, been filtered of contigs with high GC-content, making the assemblies inherently biased towards AT-richness; they were therefore excluded from all analyses in this study. Anaerobic fungi from the genus *Neocallimastix* have also been suggested to have an extreme AT-bias [48], though the species that have been sequenced, *Neocallimastix patriciarum* and *Neocallimastix californiae,* have relatively high transcriptome/CDS GC-content (37.1 and 29.6 %, respectively) [49].

**Table 1.** GC content (%) of the most AT-rich eukaryotes sequenced to date

Species	Host	Transcripts	CDS	Non-CDS*	Genome
*Plasmodium gallinaceum*	Birds	21.21	21.19	14.85	17.83
*Plasmodium ashfordi*†	Birds	21.22	na	na	na
*Plasmodium relictum*	Birds	21.60	21.57	15.27	18.33
*Plasmodium gaboni*	Primates	22.42	22.44	12.78	18.21
*Plasmodium falciparum*	Primates	23.79	23.78	14.28	19.34
*Plasmodium berghei*	Rodents	23.79	23.75	19.95	22.04
*Plasmodium yoelii*	Rodents	23.94	23.91	19.62	21.74
*Plasmodium reichenowi*	Primates	24.07	24.06	13.72	19.26
*Plasmodium vinckei*	Rodents	24.70	24.66	20.62	22.89
*Nosema apis*	Insects	na	24.83	16.64	18.78
*Plasmodium chabaudi*	Rodents	25.58	25.53	21.25	23.62
*Hepatospora eriocheir*	Crustaceans	na	25.62	20.46	22.60
*Pecoramyces ruminantium*‡	Ruminants	na	26.76	14.31	17.00
*Nosema ceranae*	Insects	27.42	27.36	24.40	25.27
*Dictyostelium discoideum*	na	27.42	27.41	14.40	22.44
*Tetrahymena thermophila*	na	na	27.53	17.24	22.32
*Entamoeba dispar*	Mammals	27.72	27.72	20.09	23.67
*Tetrahymena elliotti*	na	na	27.74	19.10	22.94
*Entamoeba nuttalli*	Mammals	27.78	27.78	21.50	25.02
*Enterocytozoon hepatopenaei*	Crustaceans	na	27.82	19.53	25.45
*Anncaliia algerae*§	Insects	27.74	27.84	21.92	23.21
*Strongyloides ratti*	Rodents	na	27.98	16.91	21.43

*Introns and intergenic sequences (non-coding).

†Data from this species are derived from a transcriptome assembly [30]

‡This species was previously known under the name *Orpinomyces* sp. C1A [57].

§This species was previously known under the name *Brachiola algerae* [37].

### Sequences

**Fig. 1.**
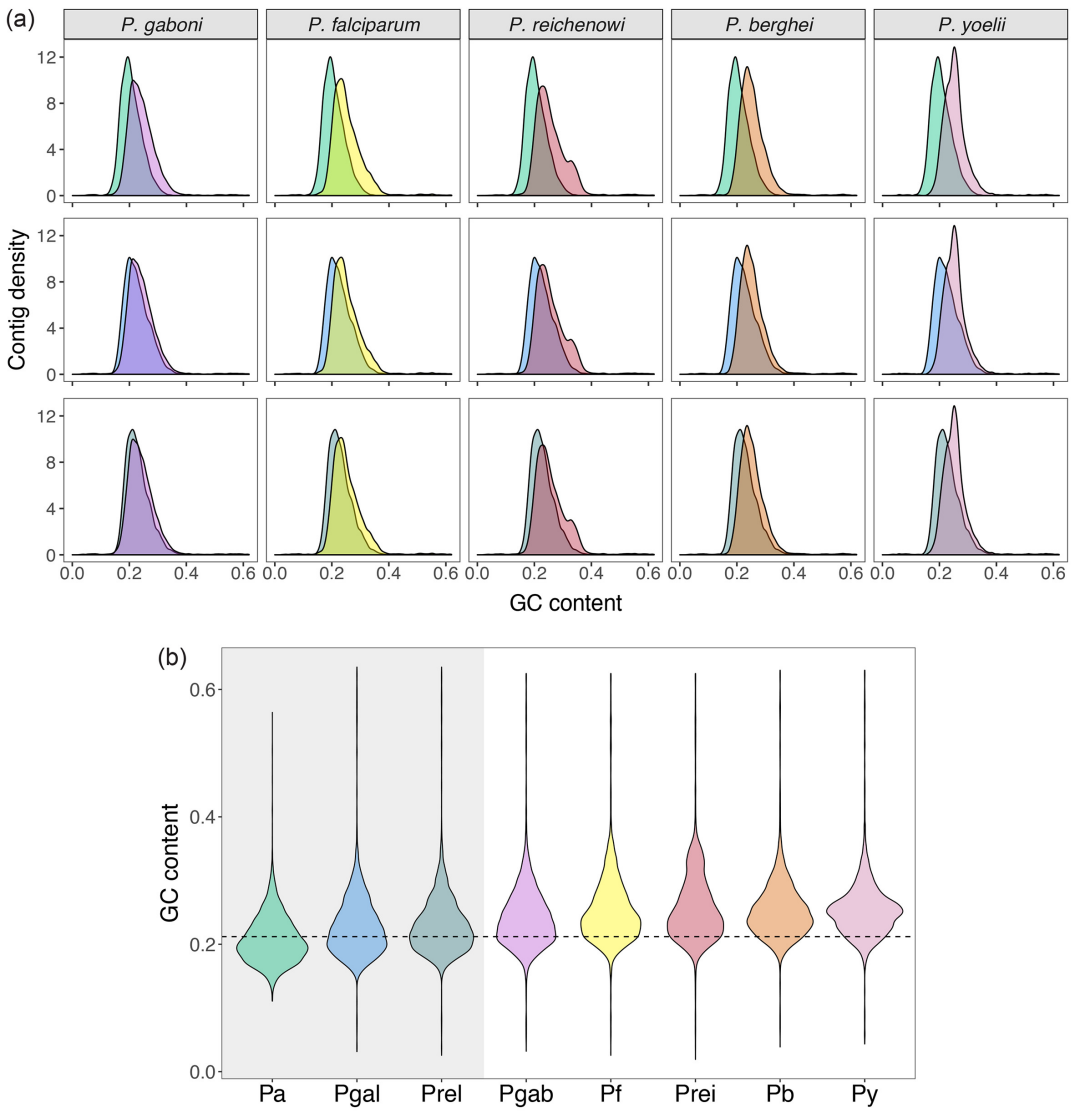
Comparative transcriptome GC-content of the eight eukaryotes with the most AT-rich genes. (a) Density GC curves of *P. ashfordi* (first row, in green), *P. gallinaceum* (second row, in blue), and *P. relictum* (third row, in turquoise). (b) Violin plot of transcriptome GC-content. The grey shaded area represents the bird-infecting malaria parasites and the dashed horizontal line shows the mean GC-content of *P. ashfordi* and *P. gallinaceum* (21.2 %). Pa, *P. ashfordi*; Pgal, *P. gallinaceum*; Prel, *P. relictum*; Pgab, *P. gaboni*; Pf, *P. falciparum*; Prei, *P. reichenowi*; Pb, *P. berghei*; Py, *P. yoelii*.

CDS represent the protein-coding regions of the genome, while transcripts can additionally include 5′ and 3′ untranslated regions (UTRs) and possibly also poly-A-tails. Non-CDS include both introns and intergenic sequences. Potential structural differences between transcripts and CDS is the reason why both of these datasets are presented in Table 1 to facilitate assessment. However, overall GC-content for all species was virtually identical between transcripts and CDS, which differed by only a few per ten thousand (‱) (Table 1). For example, *P. falciparum* differed by exactly 1‱ in GC-content between its transcripts (0.2379) and CDS (0.2378). All *Plasmodium* genome sequences included in the comparative analyses have been well-sequenced and gene annotations are of good quality. Though no species of *Plasmodium* has a genome that is entirely ‘complete’, the CDS (which are the focus of this paper) are well-assembled and highly comparable across species (Table 2). Genes from organellar genomes (such as mtDNA and apicoplast DNA) are present in all *Plasmodium* sequences included in the overall comparative GC analyses (Table 2). The transcript and coding sequences evaluated were derived directly from annotated genome assemblies, and are therefore not biased to specific life-cycle stages. *P. ashfordi* does not currently have a genome assembly, and therefore constitutes the exception, with the transcriptome obtained from the erythrocytic life stages at two time-points in three host individuals [30]. *P. ashfordi* was included in the overall GC analyses due to the limited number of non-mammalian *Plasmodium* species sequenced, showing strong correspondence in GC-content to the two other avian malaria parasites (Fig. 1), but was not included in downstream comparative analyses because of the incomplete gene sets resulting from the lack of a genome sequence. To allow for fair comparisons between datasets, all GC analyses that included *P. ashfordi* were performed using annotated transcripts, and the analyses without *P. ashfordi* utilized CDS. Gene categories of the seven eukaryotes with the most AT-rich genes (and available genome sequences) were selected with the intention to cover both highly-conserved and rapidly-evolving genes, as well as genes with documented unusual GC-content (highly-expressed and sub-telomeric genes). The CDS of gene sets, by category, were downloaded via PlasmoDB [31]. The category ‘non-orthologs to Pf’ was created by obtaining genes without orthologs to *P. falciparum* in the evaluated species; ‘orthologs in genus’ were genes in which the orthology phylogenetic profile (determined by the OrthoMCL algorithm [50]) was constrained to all species of *Plasmodium* analysed (Fig. 3); and the gene set ‘orthologs in phylum’ was constrained to all available Apicomplexa species in the orthology phylogenetic profile at PlasmoDB. The dataset ‘sub-telomeric genes’ was created by collecting all protein-coding genes located within a 50 kb distance to the telomeres in each species, and ‘highly expressed genes’ were created by obtaining orthologs in each species to the most highly-expressed genes (top 5 %) in the *P. falciparum* blood-stage transcriptome dataset produced by Otto *et al*. [51]. Amino acid composition was calculated using annotated protein sequences, graphs were made using ggplot2 [52], and analyses were performed using BEDTools [53] and R (v. 3.3.2) [54].

**Table 2.** Genome statistics of the *Plasmodium* species analysed

Species	GC (%) CDS	Genome size (Mbp)	Organellar genomes	Protein coding genes	Contigs*	Transcripts	CDS	Orthologs	Version
*P. gallinaceum*	21.19	25.03	Yes	5307	154	5439	5307	5233	2017-01-09
*P. relictum*	21.57	22.61	Yes	5178	514	5306	5178	5108	2017-01-09
*P. gaboni*	22.44	20.39	Yes	5286	833	5590	5774	5196	2016-06-16
*P. falciparum*	23.78	23.33	Yes	5460	16	5800	5734	5458	2015-06-18
*P. berghei*	23.79	18.78	Yes	5067	21	5254	5094	5067	2017-01-09
*P. yoelii*	23.91	23.08	Yes	6091	16	6258	6094	6091	2016-10-27
*P. reichenowi*	24.06	24.06	Yes	5769	372	6071	6012	5733	2015-06-18
*P. vinckei*	24.66	18.22	No	4954	49	5009	4954	4944	2014-06-17
*P. chabaudi*	25.53	18.97	Yes	5217	16	5364	5217	5216	2015-06-18
*P. vivax*	46.30	27.01	Yes	5552	2748	5631	5552	5550	2015-06-18

*Number of contigs/chromosomes making up the genome assembly, including organellar genome sequences. Example: the *P. berghei* genome assembly includes 14 nuclear chromosomes, one mitochondrial genome, one apicoplast genome, and five extra contigs with unplaced sequences [31].

## Results and discussion

### GC-content of the eukaryotes with the most AT-rich genes

The mean GC-content of all CDS/transcripts was found to be very low in several parasitic and free-living eukaryotes (Table 1), but exceptionally low in three *Plasmodium* parasites that infect birds [30, 32]: *P. gallinaceum*, *P. ashfordi* and *P. relictum* (21.2‒21.6 %). In fact, by a wide margin, these avian malaria species take the current lead as the eukaryotes with the most AT-rich gene sequences to date (Table 1 and Fig. 1). The human malaria parasite, *P. falciparum,* repeatedly designated as the organism with the most AT-rich genome sequence, has a transcriptomic mean GC-content of 23.79 %, which is significantly higher than that of the avian malaria parasites (Wilcoxon rank sum test: W=9 966 100, *P*<2.2e-16). Closely related to *P. falciparum* are two chimpanzee parasites that recently had their genomes sequenced, *P. reichenowi* and *P. gaboni* [33]. These two organisms contain a low transcriptomic GC-content as well (Table 1), although significantly higher than for example *P. ashfordi* (Wilcoxon test, *P. reichenowi*: W=9 905 000, *P*<2.2e-16, and *P. gaboni*: W=11 935 000, *P*<2.2e-16) (Fig. 1). This information challenges the commonly held view that *P. falciparum* is the most extreme eukaryote in terms of AT-bias, and facilitates future *Plasmodium* phylogenetic inferences. The evolutionary causes driving the extreme nucleotide composition in avian *Plasmodium* require further research and additional genomic resources, yet we can improve our understanding of the processes involved in genome evolution by characterizing the nucleotide composition of these AT-biased eukaryotic organisms.

Avian and reptile blood parasites have been particularly challenging to sequence because of the nucleated erythrocytes of their hosts. The three most AT-rich organisms, *P. gallinaceum*, *P. ashfordi* and *P. relictum,* therefore constitute the first non-mammalian species of the genus *Plasmodium* with genome or transcriptome sequences available. In addition, all three species were sequenced using Illumina technology [30, 32], which suffers from a well-known underrepresentation bias of AT-rich sequences due to difficulties in sequencing reads composed of homogenous bases [55, 56]. This problem was also encountered by Youssef *et al.* [43], who struggled with assembling the fungal genome of *Pecoramyces ruminantium* [57] due to low intronic GC-content. The solution to this challenge was a hybrid genome sequence assembly based on both Illumina and PacBio reads ‒ allowing the AT-rich introns to be properly assembled, leading to the lowest genome-wide GC-content so far observed in any eukaryote (17.0 %; Table 1). Because of the Illumina sequencing bias, we can suspect that fewer *Plasmodium* reads with extremely high AT-content were successfully sequenced compared to reads with higher GC-content. As a result, it is likely that the three avian *Plasmodium* species even have a slightly lower GC-content than their current calculated values show.

Intriguingly, the passerine-infecting species *P. ashfordi* displays a pattern indicating a transcriptome GC-content possibly lower than that of the chicken parasite *P. gallinaceum* (Fig. 1). It is likely that highly AT-rich sequences from *P. ashfordi* may have been filtered during the strict assembly criteria, leading to a higher mean GC-content than the true value. In fact, besides the annotated transcriptome of *P. ashfordi*, the dataset includes a smaller, unannotated transcriptome assembly [30] which encompasses a remarkably low GC-content of 17.26 % (not used in this study). Since this particular species’ GC-content is based solely on a blood-stage transcriptome, future whole-genome sequencing efforts will have to determine just how low GC-content *P. ashfordi* has evolved.

### Comparative GC analyses of subsets of genes in the most AT-rich eukaryotes

**Fig. 2.**
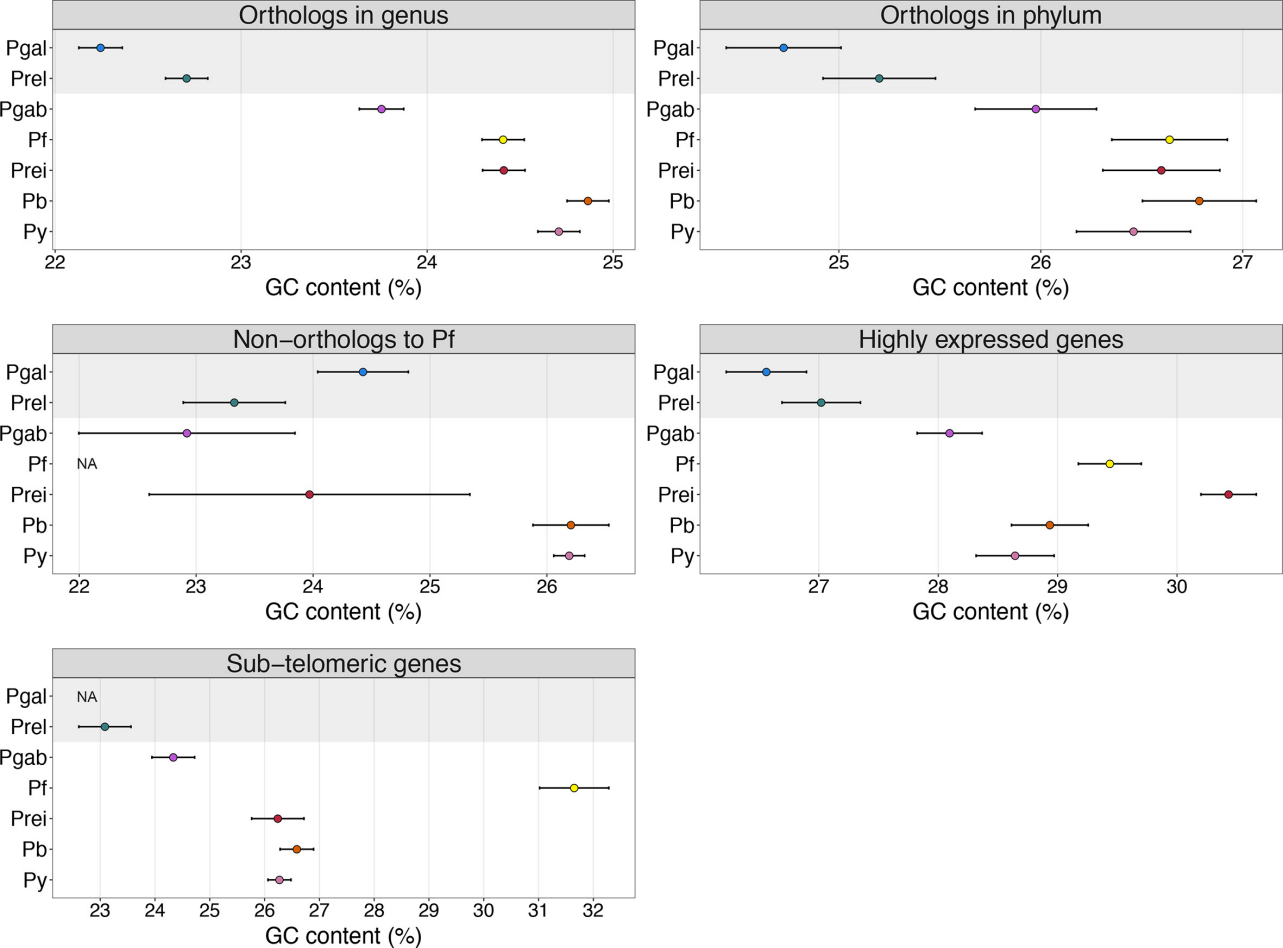
GC-content by gene category in the seven eukaryotes with the most AT-rich genes and with genome sequences available. Points signify mean GC-percentages and horizontal lines delineate the 95 % confidence interval. The shaded area represents the bird-infecting malaria parasites. Pgal, *P. gallinaceum*; Prel, *P. relictum*; Pgab, *P. gaboni*; Pf, *P. falciparum*; Prei, *P. reichenowi*; Pb, *P. berghei;* Py, *P. yoelii*.

To evaluate whether the overall pattern of AT-bias in the avian malaria parasites was skewed towards specific groups of genes or evident across multiple gene categories, comparative GC analyses of subsets of genes were performed using the seven eukaryotes with the most AT-rich genes for which a complete genome sequence was available (*P. ashfordi* was therefore not included) (Tables 2, S1 and S2, available in the online version of this article). The GC-content of all orthologs in the genus *Plasmodium* (*n*=4499‒4582) showed a strong, consistent AT-bias in the two avian parasites *P. gallinaceum* and *P. relictum* compared to the mammalian parasites *P. gaboni*, *P. falciparum*, *P. reichenowi*, *P. berghei* and *P. yoelii* (Fig. 2). Next, highly-conserved orthologs present in the phylum Apicomplexa were investigated (*n*=619‒631), and these sequences also displayed a strong AT-bias in the avian parasites (Fig. 2). Highly-expressed genes and genes located in the sub-telomeric regions of the *P. falciparum* genome have previously been shown to exhibit comparatively higher GC-content relative to other genes [4, 22, 28]. Analyses of the seven AT-rich species of *Plasmodium* showed that highly-expressed genes were indeed higher in GC-content for all species, though still comparatively lower in *P. gallinaceum* and *P. relictum* (Fig. 2). Similarly, sub-telomeric genes showed high GC-content in *P. falciparum*, and low GC-content in the avian species of the genus *Plasmodium*. Genes without orthologs to *P. falciparum* (non-conserved genes) were the only group where the avian species of the genus *Plasmodium* did not show a clear AT-bias compared to other species (Fig. 2). However, the number of genes in this category varies drastically across species (*n*=77‒1530) (Table S1), complicating any potential inferences about their nucleotide content. Additional GC comparative analyses of three *Plasmodium* multigene families involved in host–parasite interactions (with somewhat similar gene numbers across species) showed no difference in mean GC-content between avian (22.48 %) and mammalian parasites (22.23 %) for the RBP family (Wilcoxon test: W=1140, *P*=0.68) (Table S2). However, the two gene families RhopH1 and eTRAMP showed significantly reduced GC-content in the avian parasites (RhopH1=22.34 %; eTRAMP=26.32 %) relative to the mammalian parasites (RhopH1=26.35 %; eTRAMP=31.14 %) (Wilcoxon test: RhopH1, W=341, *P*=3.78e-5; eTRAMP, W=1319.5, *P*=4.01e-7) (Table S2).

### Genomic compositional constraints of avian *Plasmodium* parasites

To generate an overview of the compositional constraints the extremely AT-biased bird-infecting *Plasmodium* species must be subjected to, codon usage in both *P. gallinaceum* and *P. relictum* was investigated, and compared to that of an AT-rich congeneric (*P. falciparum*) and a GC-rich congeneric (*P. vivax*). Overall, the AT-bias in the avian parasites’ coding sequences was reflected in drastically biased codon usage (Table S3). While *P. vivax* has a diverse and heterogeneous codon usage, alternating between several codon variants for each amino acid, *P. gallinaceum* and *P. relictum* exhibit a highly homogenous usage for codons ending in adenine or thymine. This biased usage of AT-rich codons was even more pronounced than that observed in *P. falciparum* for most amino acids (Table S3).

**Fig. 3.**
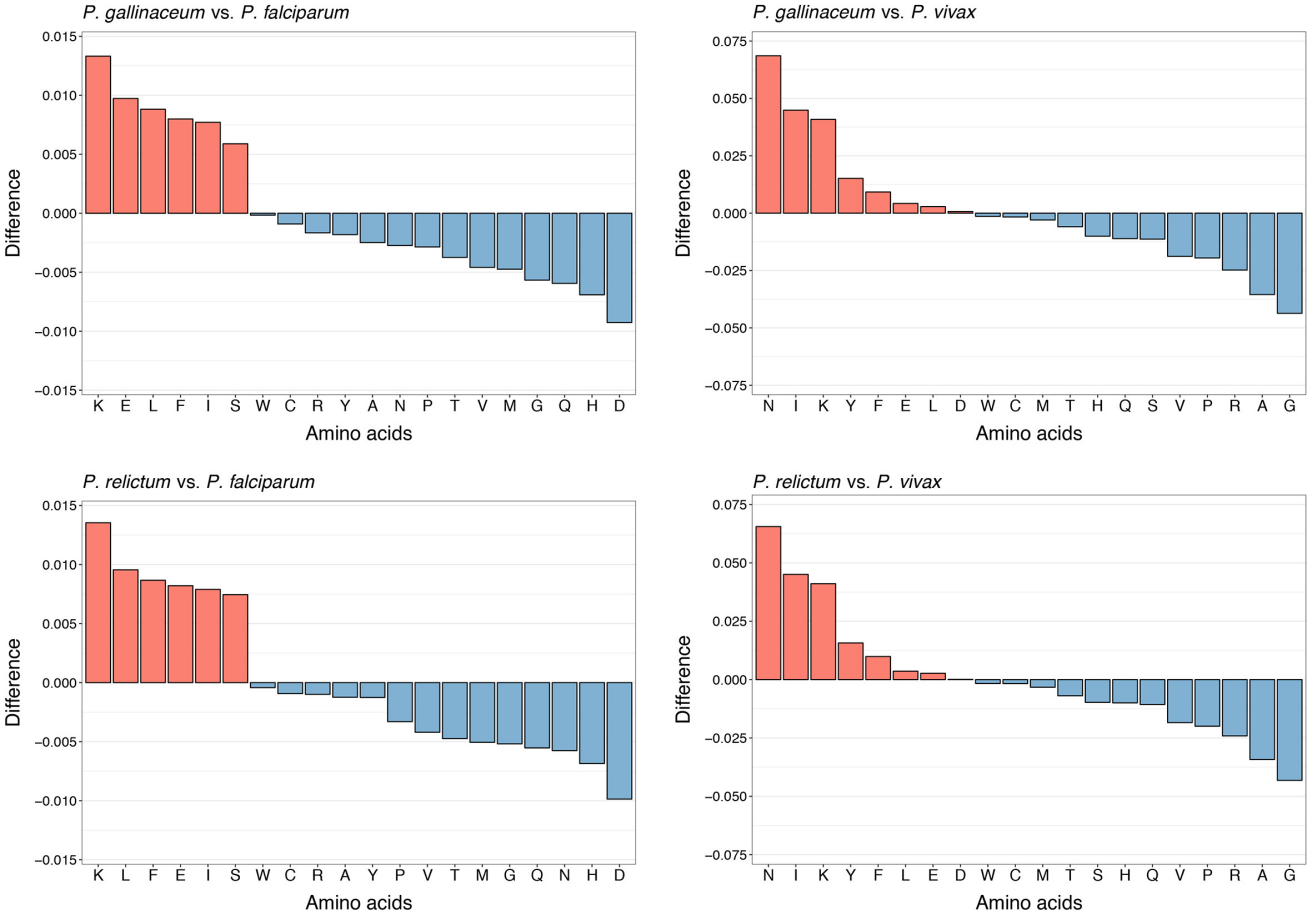
Relative pairwise differences in amino acid proportions of predicted proteins in the genomes of *P. gallinaceum* versus *P. falciparum* (an AT-rich congeneric) and versus *P. vivax* (a GC-rich congeneric). The same comparison is made for *P. relictum* versus *P. falciparum* and versus *P. vivax*. Positive values (red bars) indicate a larger relative proportion of the denoted amino acids in the genomes of either *P. gallinaceum* or *P. relictum*. Note the differences in scale in the y-axes between the *P. falciparum* and the *P. vivax* comparison.

Finally, to get a glimpse into the amino acid production of severely nucleotide constrained eukaryotes, overall amino acid proportions in the genomes of *P. gallinaceum* and *P. relictum* were compared to the human-infecting parasites *P. falciparum* and *P. vivax*. As expected, a larger difference in the proportion of amino acids was found in the comparison to the GC-rich *P. vivax* than to the AT-rich *P. falciparum* (Fig. 3). However, not only the relative scale of amino acid proportion was different, the composition of amino acids differed substantially in the comparison to *P. falciparum* versus the comparison to *P. vivax*. Compared to *P. falciparum*, lysine (K) was the amino acid with highest relative difference in the coding sequences of the avian species of the genus *Plasmodium*, while aspartic acid (D) and histidine (H) were more abundant in *P. falciparum* (Fig. 3). Compared to *P. vivax*, however, the coding sequences of the avian malaria parasites had a relative increase of asparagine (N) and a scarcity of glycine (G) and alanine (A). Both glycine and alanine are GC-rich amino acids (alanine requires a codon composed of GCN, and glycine requires a codon of GGN), so it is not surprising that these particular amino acids have declined in relative proportion in AT-biased organisms if the selection pressure to keep them intact has not been able to overcompensate mechanisms of GC→AT substitutions [19].

### Conclusion

In conclusion, this comparative genomic study shows that the eukaryotic organisms with the most AT-rich genes sequenced to date are distributed across fungi, ciliates, apicomplexans and amoebas though dominated by *Plasmodium* species. Interestingly, it is the *Plasmodium* parasites infecting birds that have evolved the most extreme coding sequences in terms of AT-bias. The genes of avian malaria parasites are subject to remarkable compositional constraints, such as biased codon usage, which makes these organisms excellent candidates for studying genomic architecture, and incites further questions about the evolutionary causes and biological consequences of extreme genomic AT-bias.

## Supplementary Data

24 Supplementary File 1

## Supplementary Data

18 Supplementary File 2
