# Supplementary material for: Plasmodium parasites of birds have the most AT-rich genes of eukaryotes
Source: Microb Genom. 2018 Jan 23;4(2):e000150. doi: 10.1099/mgen.0.000150 (PMC5857377; doi:10.1099/mgen.0.000150)
Supplement: Supplementary File 1 [file mgen-4-150-s001.pdf]

Table S1. Mean GC content (%) and number of genes of different gene categories in seven *Plasmodium* species. This information is an extension of Figure 2.

Species	Host	Orthologs in genus	Non-orthologs to Pf	Conserved orthologs in phylum	Highly expressed genes	Sub-telomeric genes
P. gallinaceum	Birds	20.99 (n=4499)	21.67 (n=591)	23.80 (n=631)	25.79 (n=558)	na
P. relictum	Birds	21.54 (n=4512)	21.08 (n=470)	24.28 (n=630)	26.33 (n=559)	21.74 (n=283)
P. gaboni	Primates	22.24 (n=4582)	21.16 (n=128)	24.64 (n=625)	27.26 (n=869)	23.28 (n=333)
P. falciparum	Primates	23.01 (n=4578)	na	25.30 (n=626)	30.21 (n=1047)	33.59 (n=177)
P. reichenowi	Primates	22.96 (n=4547)	22.61 (n=77)	25.20 (n=628)	31.14 (n=1336)	26.34 (n=335)
P. berghei	Rodents	23.58 (n=4542)	26.04 (n=505)	25.66 (n=619)	27.92 (n=530)	25.74 (n=349)
P. yoelii	Rodents	23.51 (n=4558)	26.27 (n=1530)	25.33 (n=623)	27.64 (n=531)	26.48 (n=381)

Table S2. Mean GC content (%) of three *Plasmodium* multigene families involved in host-parasite interactions.

Species	Host	eTRAMP	RBP	RhopH1/ CLAG
P. gallinaceum	Birds	26.61 (n=12)	21.89 (n=14)	23.71 (n=4)
P. relictum	Birds	26.58 (n=14)	22.51 (n=33)	22.19 (n=9)
P. gaboni	Primates	30.84 (n=13)	20.26 (n=8)	26.34 (n=15)
P. falciparum	Primates	30.46 (n=14)	20.85 (n=7)	25.94 (n=5)
P. reichenowi	Primates	30.64 (n=14)	21.29 (n=7)	26.20 (n=6)
P. berghei	Rodents	32.11 (n=7)	22.81 (n=15)	28.32 (n=2)
P. yoelii	Rodents	31.17 (n=12)	23.23 (n=14)	28.25 (n=2)

eTRAMP = early transcribed membrane protein, RBP = reticulocyte binding protein, CLAG = cytoadherence-linked asexual gene, RhopH1 = high molecular mass rhoptry protein 1.
